# From economic visions to healthier nations: the role of Gulf Cooperation Council countries national development visions in shaping noncommunicable disease policies

**DOI:** 10.3389/fpubh.2026.1899157

**Published:** 2026-07-29

**Authors:** Rasha Alfawaz, Nada AlNaji, Ghadeer S. Aljuraiban, Najla Alorayyidh, Hussain Al Rand, Samya Ali Bahram, Riyad Qainan Alghamdi, Zakariya Yahya Al Balushi, Mohammed Al Thani, Al Munther Al Hasawi, Pasi Penttinen

**Affiliations:** 1 Public Health Programs and Policies, Gulf Center for Disease Prevention and Control, Riyadh, Saudi Arabia; 2 Department of Community Health Sciences, College of Applied Medical Sciences, King Saud University, Riyadh, Saudi Arabia; 3 Ministry of Health and Prevention, Dubai, United Arab Emirates; 4 Ministry of Health, Manama, Bahrain; 5 Public Health Authority, Riyadh, Saudi Arabia; 6 Ministry of Health, Muscat, Oman; 7 Ministry of Public Health, Doha, Qatar; 8 Ministry of Health, Kuwait City, Kuwait

**Keywords:** development plans, Gulf Cooperation Council, health policy, multisectoral coordination, noncommunicable diseases, sustainable development, vision

## Abstract

**Background:**

Noncommunicable diseases (NCDs) account for about 73.7% of deaths across the Gulf Cooperation Council (GCC). GCC states have adopted long-term national development visions to provide a coherent framework for economic diversification. A synthesis of how these visions shape NCD policy priorities is needed to understand their role as action-guiding frameworks and identify opportunities for continued system strengthening.

**Methods:**

We conducted a qualitative policy analysis using an interpretive approach, covering six vision documents, the Gulf Center for Disease Prevention and Control (Gulf CDC) NCD policy mapping report, and 18 key-informant interviews from health and non-health sectors across GCC countries. We coded documents and transcripts and synthesized findings across themes.

**Results:**

Vision documents positioned health as a development priority and referenced prevention, healthy lifestyles, or health-system reform. Measurable targets varied, with some frameworks specifying indicators such as life expectancy or premature NCD mortality. Stakeholders reported that visions elevated NCDs on political agendas and facilitated multisector measures including food-reformulation, Healthy Cities initiatives, and expanded NCD screening. They also highlighted areas where continued system strengthening could support more consistent translation into sectoral programs.

**Conclusion:**

GCC development visions can function as high-level levers for NCD prevention and health-system reform when supported by cross-sector coordination, clear indicators, and routine monitoring and evaluation.

## 1 Introduction

Noncommunicable diseases (NCDs), particularly cardiovascular diseases (CVDs), diabetes, cancers, and chronic respiratory illnesses, are leading causes of mortality across Gulf Cooperation Council (GCC) countries (the United Arab Emirates (UAE), Bahrain, Saudi Arabia, Oman, Qatar, and Kuwait), accounting for 73.7% of all deaths (1, 2). Their health and economic costs are substantial, estimated at $16.7 billion annually (2). GCC governments have therefore recognized that addressing NCDs is critical for public health and the sustainability of economic development.

All six GCC states have launched national development vision plans since the mid-2000s. Governments use these visions as top-level strategic frameworks to set priorities, align ministries around shared targets, and guide reforms. This makes them relevant to NCD prevention because many determinants of NCD risk sit outside the health sector, including food systems, urban design, transport, education, and employment (3, 4). Vision documents outline long-term goals, typically to 2030 or beyond, that aim to diversify economies away from hydrocarbons, expand private-sector-led growth, strengthen human capital, and modernize service delivery (5–10). These include the UAE’s “We the UAE-2031,” which constitutes a comprehensive federal reform agenda, aligned with the UAE Centennial 2071 (5), Bahrain’s Economic Vision 2030 (10), Saudi Vision-2030 (8), Oman Vision-2040 (6), Qatar National Vision-2030 (9), and Kuwait Vision-2035 (also called “New Kuwait”) (7). While primarily economic, they encompass social development pillars such as education and health (5–10).

These vision frameworks sit within the broader 2030 Agenda for Sustainable Development and its Sustainable Development Goals (SDGs), which many governments use to frame national priorities and indicators (11). In the health domain, SDG target 3.4 commits countries to reduce premature mortality from NCDs by one third by 2030 through prevention and treatment (12).

GCC visions generally present health as both a societal goal and an economic enabler. They emphasize prevention and healthy lifestyles as pillars for good health. Many also place health within broader “quality of life” agendas, linking well-being to social development and economic performance. For example, the UAE’s “We the UAE-2031” frames health under its social empowerment agenda and calls for “an innovative, state-of-the-art healthcare system” that enhances quality of life and supports healthy lifestyles. Qatar’s Vision 2030 (9) aspires to “a healthy population, physically and mentally” and an integrated healthcare system of world-class standard. Saudi Arabia’s Vision 2030 (8) includes “Living Healthy, Being Healthy” under its Vibrant Society pillar, emphasizing that a “healthy lifestyle is an essential mainstay of a high quality of life.” Bahrain’s Vision 2030 (10) aims for accessible, high-quality healthcare for all and pledges to “address the key risk factors” affecting population health by promoting healthy lifestyles and preventive care. Bahrain was among the first GCC countries to integrate health-system sustainability and reduction of non-communicable disease (NCD) risk factors within a national development framework.

Several GCC visions are approaching their 2030 milestones, and governments have entered monitoring and revision phases. This creates a timely opportunity to examine what these high-level strategies have meant for NCD policy in practice. Synthesized evidence across all six GCC countries remains limited, making transferable lessons and implementation features difficult to identify. To our knowledge, few studies have triangulated vision texts, cross-GCC policy mapping, and multisector stakeholder accounts to describe mechanisms linking visions to NCD policy delivery. This paper complements a recent GCC-wide NCD policy-mapping project coordinated through the Gulf Center for Disease Prevention and Control (Gulf CDC). Established in 2022, Gulf CDC provides a regional platform for shared evidence generation and peer learning. It can also support policy harmonization where regional consistency matters, such as taxation, food standards, and tobacco import regulation, helping countries align approaches and reduce cross-border implementation barriers. Focusing on national development visions as centrally led, whole-of-government frameworks, we document how visions frame NCD prevention, shape governance and multisector pathways, and inform regional coordination, including Gulf CDC’s role as a policy-alignment platform. We therefore review the six GCC visions through the lens of NCD policy, synthesizing evidence on how they shape NCD policy priorities and governance.

## 2 Methods

### 2.1 Study design

**Figure 1:**
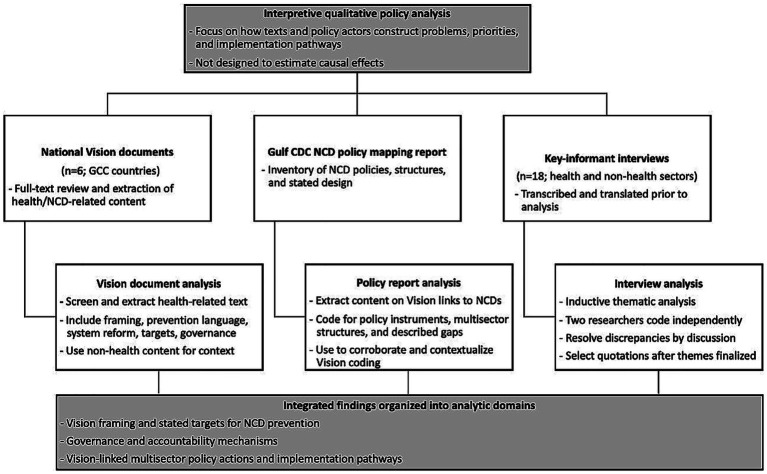
Methods workflow and triangulation across vision documents, the Gulf CDC NCD policy mapping report, and key-informant interviews.

We conducted a qualitative policy analysis using an interpretive approach. Consistent with interpretivism, we examined how national vision documents and stakeholders described NCD priorities, governance, and implementation pathways. We did not aim to test whether national visions caused changes in NCD outcomes. Instead, we analyzed how visions were used to frame NCD prevention, support multisectoral action, and guide policy priorities. Some co-authors also hold decision-making and policy-forming roles in the region, including members of the Gulf CDC Supervisory Committee, comprising senior public health officials representing the six GCC countries. We explicitly acknowledged this dual positionality within our reflexive process, using team discussion and triangulation to challenge assumptions and check interpretations. We analyzed vision documents, findings of the GCC-wide NCD policy mapping project (13), and key-informant interviews conducted for that project. Together, these sources were used to understand the interplay between national development visions and NCD policy in the GCC context. The analysis covered all six GCC countries. Figure 1 summarizes the methods workflow and shows how we triangulated evidence across vision documents, the Gulf CDC mapping report, and key-informant interviews.

### 2.2 Data sources

#### 2.2.1 National vision documents

We obtained the primary national vision strategy documents for each country, including: We the UAE-2031 (5), Bahrain Economic Vision-2030 (10), Saudi Vision-2030 (8), Oman Vision-2040 (6), Qatar National Vision-2030 (9), and New Kuwait-2035 (7). These were sourced from government websites and publications. We reviewed each vision document in full and extracted all content related to health, healthcare system reform, NCD prevention, or wellness promotion. We focused on stated health objectives, priority areas (e.g., lifestyle, preventive-care), and targets relevant to NCD outcomes (e.g., life expectancy, disease prevalence, risk reduction).

#### 2.2.2 Gulf-CDC NCD policy mapping report

We reviewed the “Mapping of NCD Policies in the GCC Region” main report (2025) by the Gulf CDC. The report provides a cross-country inventory of NCD-related policies, strategies, and initiatives, including analysis of how national policies align with global best practices and development frameworks. Like other policy mapping exercises, it primarily describes the existence and stated design of policies and structures, and may not fully capture implementation or effectiveness. It may also under-represent unpublished or subnational initiatives.

As part of the mapping project, structured key-informant interviews were conducted, mostly in Arabic, with stakeholders in each GCC country, primarily senior officials and experts with NCD policy roles. Interviews were transcribed and translated into English. There were 2–4 interviewees per country, totaling 18 stakeholders from the health sector, including Ministries of Health and public health authorities, and relevant non-health sectors, including education, agriculture, municipal affairs, and youth. The interviews explored political commitment to NCDs, intersectoral collaboration, opportunities for system strengthening, the role of national development plans, and views on regional collaboration, including the Gulf CDC’s role in supporting national NCD efforts. Ethical approval and consent were obtained for the original study.

### 2.3 Data analysis

We used an iterative, integrative approach to synthesize evidence from three sources; vision documents, Gulf CDC policy mapping report, and key-informant interview transcripts. We first analyzed each source type separately, then integrated findings through triangulation during interpretation. This approach allowed us to distinguish what is stated in high-level strategy documents from what was reported as implemented, and from stakeholder perspectives on how visions shaped policy processes. For example, when vision documents articulated priorities (prevention or regulatory reform), we examined the mapping report for corresponding policy instruments or structures and examined interview materials for reported pathways of influence, implementation, and perceived constraints. This integrated step is interpretive and explanatory in the limited sense that it identifies plausible mechanisms and enabling conditions described by stakeholders. It does not test causal effects of visions on NCD outcomes.

#### 2.3.1 Vision document analysis

We reviewed each national vision document in full. Screening was conducted using keyword-assisted full-text searching followed by manual review of relevant sections. Search terms included: health, healthcare, public health, prevention, wellness, well-being, quality of life, healthy lifestyle, NCD, noncommunicable disease, chronic disease, cardiovascular disease, diabetes, cancer, respiratory disease, obesity, hypertension, risk factor, tobacco, smoking, physical activity, nutrition, diet, food, sugar, salt, sodium, trans fat, screening, early detection, mortality, life expectancy, target, indicator, monitoring, evaluation, governance, multisectoral, intersectoral, whole-of-government, Health in All Policies, and accountability. Where Arabic versions of documents were reviewed, equivalent Arabic terms were used.

Two researchers independently screened the documents and extracted relevant text into a matrix. Extracted text was then compared, and differences were resolved through discussion with the wider study team. The predefined coding categories were: health framing; prevention and risk-factor language; health-system reform and service delivery; measurable targets and indicators; governance, accountability, and cross-sector roles; and regional coordination or policy alignment. Text not directly related to health was retained only when it provided context for cross-sector determinants of NCD policy, such as economic transformation, urban planning, education, or public-sector performance.

The determinant categories were screened explicitly. Using the wider determinants framing, the vision-linked actions identified in the analysis were grouped into downstream behavioral and biological risk-factor policies, midstream setting-based interventions, and upstream governance and economic measures (14). Downstream factors included diet and nutrition, salt, sugar, trans fats, tobacco use, physical inactivity, obesity, hypertension, dyslipidemia, diabetes, screening, and early detection. Midstream determinants included schools, workplaces, health care access, insurance, community settings, Healthy Cities, housing, transport, walkability, and urban planning. Upstream determinants included national development priorities, economic policy, taxation, regulation, food standards, governance, interministerial coordination, financing, monitoring systems, and accountability. These categories were used as sensitizing concepts rather than as rigid codes, allowing the analysis to capture both explicit NCD language and broader policy mechanisms that could plausibly affect NCD prevention.

#### 2.3.2 Policy mapping report analysis

We treated the Gulf CDC policy mapping report as a secondary source documenting the presence and stated design of NCD-related policies, strategies, initiatives, and governance arrangements across countries. We extracted and coded content that referenced linkages between visions and NCD policy, including vision-aligned strategies, multisectoral structures, and described gaps. This coding captured whether initiatives were described as aligned with vision priorities, regardless of effectiveness, coverage, or measured impact, which we did not assess. We used the report to corroborate and contextualize the vision document findings and identify examples of policy actions described as connected to vision priorities.

#### 2.3.3 Interview analysis

The interviews were conducted as part of the Gulf CDC NCD policy mapping project (see Supplementary Table 1 for the interview guide). A structured interview guide with open-ended questions and optional probes was used, rather than unstructured interviews. The guide covered institutional roles in NCD prevention, cross-sector engagement, policy development and implementation, political commitment and national development visions, barriers and enablers, success stories, monitoring and evaluation, and views on the Gulf CDC’s regional role. Example questions included: “What is your institution’s role in the prevention and control of CVD, diabetes, and related risk factors?,” “Which ministries or sectors are involved in policy development or implementation?,” “How have national visions or political commitments shaped NCD policies?,” “What are the main barriers and enablers to implementation?,” and “How can the Gulf CDC support national and regional NCD prevention?”

To standardize data collection, the Gulf CDC team appointed one trained interviewer to conduct the interviews, with two Gulf CDC team members attending each interview to ask follow-up questions when needed. Most interviews were conducted in Arabic, with two conducted in English, according to participant preference. Interviews were recorded, transcribed, and translated into English for analysis. In the parent mapping project, transcripts were imported into NVivo version 15 and coded using a combined deductive and inductive approach. Deductive codes were informed by policy analysis and implementation frameworks, while inductive coding captured emerging themes not covered by the initial framework. Two researchers independently re-reviewed the transcripts and coded outputs, focusing on how national visions were perceived to shape NCD agenda-setting, multisectoral governance, and implementation pathways. Differences in interpretation were resolved through discussion, and illustrative quotations were selected after themes were finalized.

## 3 Results and discussion

### 3.1 Analytic approach

We organized the integrated findings around three analytic domains: (1) how visions frame NCD prevention, (2) governance and accountability arrangements described as translating vision priorities into cross-sector action, and (3) stakeholder-reported policy actions described as occurring in the context of, or justified through, vision commitments.

Our central analytical argument is that national visions appear to function less as stand-alone “health policies” and more as high-level policy signals that can strengthen NCD prevention when they are translated through sectoral plans, programs, regulatory instruments, and cross-government coordination mechanisms. Their practical contribution appears strongest when prevention priorities are linked to measurable indicators, roles are clarified across sectors, and follow-up arrangements support learning and system strengthening. Because visions are strategic frameworks, our analysis does not assess them as legally binding implementation instruments.

### 3.2 Vision framing and stated targets for NCD prevention

We summarized the cross-cutting themes and subthemes that emerged across the vision documents, Gulf CDC mapping report, and stakeholder interviews (Table 1). The themes helped explain how national visions were linked to NCD policy priorities, governance arrangements, implementation pathways, and monitoring gaps.

**Table 1:** Integrated themes and subthemes from the document and interview synthesis.

Theme	Subthemes	Interpretation
Health as a national development priority	Health as quality of life; prevention; human capital; economic sustainability; SDG alignment	National visions appear to move NCD prevention from a health-sector issue to a whole-of-government development priority.
Governance as the translation mechanism	National committees; Health in All Policies; central reporting; legal endorsement; cross-ministry roles	Visions alone do not implement policy. Their effect depends on committees, reporting systems, and assigned responsibilities.
Multisectoral implementation beyond the Ministry of Health	Food reformulation; taxation; school health; urban planning; Healthy Cities; tobacco control; physical activity; media	NCD prevention depends on sectors that shape food, transport, schools, taxation, urban design, and communication.
Monitoring and measurable indicators	Baselines; targets; routine reporting; national surveys; data quality; accountability	Visions are more useful when they include measurable NCD indicators and reporting systems.
Implementation barriers	Staff turnover; unclear budgets; weak handover; private-sector resistance; fragmented data; limited evaluation	The main weakness is not policy absence, but implementation continuity, funding, data systems, and accountability.
Community and private-sector engagement	Public consultation; workshops; NGO participation; school-based work; community settings; Healthy Cities	Community and private-sector engagement helped translate national priorities into setting-specific action.
Regional coordination and Gulf CDC role	Harmonized regulations; shared evidence; joint awareness campaigns; capacity building; peer learning	Gulf CDC can strengthen policy alignment across countries with similar risk profiles and shared markets.

Across vision documents, health was framed as part of national development, with emphasis on shifting from curative care toward prevention, healthy lifestyles, risk factor reduction, and health-system transformation, including quality-of-care improvement and integration. This positions NCD prevention as a cross-government issue, beyond Ministry of Health responsibility.

In several vision strategies, health and NCD prevention were framed as integral to national development (2, 8, 9), contrasting with earlier responses that often treated NCD prevention mainly as a health-sector task and struggled to secure sustained whole-of-government commitment (15). The visions link health to economic sustainability through the health and financial burden of NCDs and productivity gains from prevention (9, 10), and to social progress by presenting a healthy population as a foundation for human development and workforce capability (2, 15).

A key cross-country difference is whether visions include measurable health or NCD targets. Supplementary Table 2 lists examples of targets or indicators in vision frameworks and vision-linked strategies, whereas Table 2 focuses on vision framing and perceived agenda setting. Interviewees suggested that general vision language has greater operational value with measurable indicators and routine follow-up. Bahrain and Qatar launched their visions in 2008, which interviewees described as allowing more time for institutionalization, whereas Oman Vision 2040 is more recent. These timelines may shape operationalization and performance follow-up maturity (Supplementary Figure 1 provides a temporal context).

**Table 2:** Vision framing for NCD prevention and stakeholder-reported agenda-setting role.

Country, Vision framework	Vision framing relevant to NCD prevention (document synthesis)	Stakeholder-reported agenda-setting role of the Vision (interviews)	Illustrative stakeholder quote
United Arab Emirates, “We the UAE 2031”	Frames health and quality of life as a national development aim, with emphasis on prevention, healthy lifestyles, and a future-ready system (including innovation).	Reported to elevate prevention as a cross-government priority and reinforce continuity of NCD attention across successive national agendas.	“Managing diabetes and reducing CVD mortality… have been incorporated into the UAE Vision 2031 and 2071…” By Stakeholder, UAE (Ministry of Health and Prevention)
Bahrain, Economic Vision 2030	Frames access to quality care and health-system sustainability, alongside healthy lifestyles and prevention.	Reported to provide a whole-of-government “permission structure” for prevention-oriented work, and to make cross-government cooperation easier when initiatives can be framed as Vision-aligned.	“Bahrain Vision 2030 has contributed to enhancing policies for the prevention of NCD such as the National NCD Strategy, the salt reduction initiative, and the expanded screening programs” “As long as it aligns with the goals of the vision, everyone facilitates it…” By Stakeholder, Bahrain (Ministry of Health)
Saudi Arabia, Saudi Vision 2030	Frames prevention and healthy lifestyle promotion under Vision 2030’s “Vibrant Society” pillar, which links a healthy lifestyle to quality of life and connects this to the Health Sector Transformation Program.	Reported to act as a political mandate that accelerates reforms, helps agencies align around national priorities, and supports movement on policies that require cross-sector agreement.	“Vision 2030 has been the primary foundation for significant changes… the social and health determinants are highly interconnected in controlling NCDs” By Stakeholder, Saudi Arabia (Saudi Food and Drug Authority)
Oman, Oman Vision 2040	Frames a “healthy society,” prevention, and shared responsibility across sectors, alongside health-system performance and equity.	Reported to add accountability and momentum to implement existing NCD priorities, with an expectation that ministries will respond to vision-led monitoring.	“[Vision leaders] monitor what is happening and question why something has not been done… I expect it will have a positive impact on implementation…” By Stakeholder, Oman (Ministry of Health)
Qatar, Qatar National Vision 2030	Frames health as a core pillar of human development and sustainable development, with prevention and healthy living as central themes.	Reported to raise the political profile of NCD prevention and provide high-level endorsement that strengthens multisector engagement beyond the health sector.	“This commitment has also fostered an environment where preventive care is prioritized, encouraging collaborations across sectors, and enhancing public awareness campaigns.” By Stakeholder, Qatar (Primary Health Care Corporation)
Kuwait, New Kuwait 2035	Frames health-system improvement and national development, including reduction of NCD burden as a national concern.	Reported to support policy continuity through the national development plan, including perceived stability for prevention priorities despite political fluctuations.	“The development plan is legislated by law… there is stability with regards to NCD prevention” By Stakeholder, Kuwait (Ministry of Health)

The vision synthesis comes from document coding. The quotations come from the Gulf CDC mapping project interviews and reflect perceived links to NCD prevention. Acronyms: NCDs: noncommunicable diseases; CVD: cardiovascular disease; SFDA: Saudi Food and Drug Authority; UAE: United Arab Emirates.

### 3.3 Translating visions into action: governance and accountability mechanisms

A “translation step” emerged between vision intent and implementation, often through governance structures for NCD prevention. Here, “governance structures” refers to formal bodies or processes that coordinate ministries and agencies around shared NCD priorities, including high-level councils, inter-ministerial committees, or central planning frameworks that set priorities, assign roles, and support cross-sector monitoring (3).

This aligns with WHO guidance in the Global NCD Action Plan, which encourages national multisectoral mechanisms to support policy coherence, mutual accountability, and Health in All Policies implementation (16). Interviewees’ descriptions of councils, committees, and working groups mapped onto this recommendation, while showing variability in mandate and resourcing.

Two broad patterns emerged. First, Bahrain and Qatar described health sector anchored convening bodies, such as councils or committees convening senior officials. Second, the UAE and Saudi Arabia described whole-of-government planning and executive-linked structures, including Prime Minister’s Office monitoring of NCD-related indicators within the UAE National Agenda 2021 framework and Saudi Ministerial Committee arrangements for Health in All Policies, both intended to accelerate cross-government decisions (17, 18). In Oman and Kuwait, interviewees more often described reliance on national planning councils and development plan frameworks to engage ministries outside health.

Interviewees also stressed that structures matter most when they come with sustained political backing and routine coordination. A Saudi participant noted *“Health in All Policies has a significant role… when it comes to government entities, [we have] the Public Health Authority and the ministerial committee… harmonizing with other sectors to reduce chronic diseases”* (Saudi Food and Drug Authority, Saudi Arabia). In Qatar, an official similarly noted that *“the government’s endorsement of the National Health Strategy 2018–2022 has provided a robust framework for implementing effective NCD policies”* and that this commitment *“has also fostered an environment where preventive care is prioritized, encouraging collaborations across sectors, and enhancing public awareness campaigns”* (Primary Health Care Corporation, Qatar). These arrangements can formalize authority and accountability for NCD action, but their contribution depends on collaboration, resourcing, learning, and continuity. Supplementary Table 2 summarizes governance mechanisms.

### 3.4 Vision-linked multisectoral policy actions and implementation pathways

Several categories of multisector action were described as occurring during vision periods and framed as aligned with, supported by, or justified through vision commitments. Table 3 summarizes reported implementation arrangements and illustrative actions from stakeholder interviews and the Gulf CDC policy mapping report.

**Table 3:** Translating visions into multisector action: reported implementation arrangements and illustrative actions.

Country	Reported delivery arrangement for multisector implementation (interviews and mapping report)	Illustrative multisector actions described during vision periods (examples)	Illustrative stakeholder quote
United Arab Emirates	Multisector NCD teams coordinated through the Ministry of Health and Prevention, reported to include multiple ministries and non-state partners.	Joint action planning across sectors, examples described include school-based prevention activities, municipal food environment actions (e.g., menu labelling), and multi-partner screening and awareness campaigns. Interviewees also cited innovation initiatives (e.g., digital health, biotech) aimed at a future-ready health-system and emergency preparedness.	“All relevant entities are participating… I think it is comprehensive” By Stakeholder, UAE (Ministry of Health and Prevention)
Bahrain	Prevention work coordinated through MOH public health and nutrition functions, with oversight and cross-government linkage.	Food environment work described through salt reduction in bread, with phased implementation and compliance monitoring, involving WHO technical input and domestic regulatory and inspection roles. The National Chronic Disease Management Program. Tobacco control legislative measures aligned with WHO FCTC.	“Strong leadership support, and support in budgeting” By Stakeholder, Bahrain (Ministry of Health)
Saudi Arabia	Health in All Policies ministerial arrangements and linked technical committees described as supporting cross-government review and approval of NCD-related policies.	Regulatory and standards pathway described for food-related prevention policies (for example standards on salt, sugar, fat), with technical development and cross-sector enforcement roles once approved.	“We have national committees… then a preparatory committee, then the board… once approved… [the policy] is implemented across all sectors” By Stakeholder, Saudi Arabia (Public Health Authority)
Oman	A multisector NCD committee and working groups described as organizing implementation, with vulnerability when legal authority, budgets, or staffing are limited.	Multisector engagement described through advocacy visits to other ministries and risk-factor working groups (tobacco, diet, physical activity), with sustainability challenges reported after coronavirus disease 2019.	“There needs to be an overarching framework responsible for overseeing and [tracking implementation of indicators across ministries] … The role of this [central government] is not only to fund activities but also to monitor them” By Stakeholder, Oman (Ministry of Health)
Qatar	Ministry of Public Health leadership, working through inter-ministerial committees and municipal partners, was described as enabling implementation aligned with vision priorities.	Built environment and Healthy Cities work described, including health considerations integrated into planning, plus reported nutrition-related prevention changes framed as part of prevention-oriented reform.	“Ministry of Municipality and Environment… works on creating health-promoting environments through urban planning and public health policies.” By Stakeholder, Qatar (Primary Health Care Corporation)
Kuwait	Actions are embedded in the national development plan and monitored through executive-level planning processes, with Healthy Cities described as a cross-sector coordination platform.	Healthy Cities used as a delivery platform for intersectoral collaboration, with examples described involving municipal and housing-sector contributions to health-related development plan aims.	“Healthy Cities was a very good example as a hub for intersectoral collaboration… monitored by the Cabinet” By Stakeholder, Kuwait (Ministry of Health)

MOH, Ministry of Health; NCDs, Noncommunicable Diseases; UAE, United Arab Emirates; WHO, World Health Organization.

Through the inductive thematic analysis of stakeholder interviews, and subsequent triangulation with the vision documents and Gulf CDC policy mapping report, reported actions were grouped into four main thematic areas: food environment policies, built environment and Healthy Cities initiatives, screening and service delivery reforms, and digital delivery models. These themes reflected the main pathways through which stakeholders described national visions being translated into NCD-related action. Implementation maturity varied across the six GCC states.

Our findings highlight that multisectoral action is both essential and challenging for NCD control (16). National visions were described as a unifying reference point that can help secure buy-in from non-health sectors when prevention initiatives are framed as contributing to vision priorities. The most frequently engaged sectors included education (school health and nutrition), municipalities and urban planning (Healthy Cities initiatives, walkability), finance or commerce (taxation and pricing policies, food standards), media (health promotion), youth and sports (physical activity initiatives), interior (tobacco enforcement and road safety), and environment (environmental health and related risk factors).

Illustrative cases in this study show how national and regional governance structures supported multisectoral NCD action. For example, tobacco excise taxation illustrates the role of fiscal policy and regional coordination. In 2016, GCC countries agreed to implement a harmonized excise tax on tobacco products at 100% of the pre-tax price, showing how a shared regional fiscal measure can support NCD prevention while requiring coordination beyond ministries of health, including finance, tax, customs, trade, and regulatory authorities (19). In addition, food-environment policies show how implementation depends on phased regulation, industry engagement, and monitoring. In Bahrain, salt reduction in bread involved evidence generation with WHO, regulation of bakery salt content, collaboration with laboratories and the Food Safety Department, engagement with the Ministry of Industry and Commerce and the Chamber of Commerce, and flexible traffic-light monitoring for bakeries. In Saudi Arabia, the elimination of industrially produced trans fatty acids was implemented through sequential steps, including nutrition labelling, limits on trans-fat content, voluntary private-sector pledges, and later mandatory elimination, with stakeholder engagement used to reduce disruption to the food supply chain. These examples suggest that progress can occur when high-level vision commitments are translated into specific regulatory tools, phased implementation plans, clear institutional roles, and routine monitoring. They also show that momentum is harder to sustain when governance mandates, resourcing, stakeholder engagement, and follow-up systems are weak.

### 3.5 Early outcomes and evidence of progress

To strengthen the public health grounding of the synthesis, we added selected epidemiological and implementation indicators from the Gulf CDC policy mapping report and stakeholder interviews. Early examples of policy implementation and reported progress are summarized in Supplementary Table 3, while broader public health indicators and epidemiological findings for each GCC country are summarized in Supplementary Table 4. These indicators include regional NCD burden, vision-linked mortality or risk-factor targets, screening and early-detection activities, food reformulation measures, tobacco and sugar-related policies, Healthy Cities certification, and examples of routine monitoring. Together, these tables show that the vision-linked pathways described in this study were not limited to broad strategic language, but were accompanied in several countries by measurable indicators or implementation milestones. These findings support the relevance of national visions as enabling frameworks for NCD prevention, while remaining descriptive. They do not establish that visions caused observed changes in NCD outcomes, which would require longitudinal evaluation with comparable baseline and follow-up data.

### 3.6 Regional coordination and the role of gulf CDC

The analysis highlighted Gulf CDC as a regional enabler for public health policy harmonization, shared evidence generation, capacity building, and peer learning. Gulf CDC creates an opportunity to harmonize shared economic and commercial efforts, such as taxation, food standardization, and tobacco import regulations (15). Regional alignment may also mitigate industry challenges if GCC countries adopt similar taxes or regulations. Interviewees suggested that a unified Gulf voice on NCDs could influence global health policy, especially on challenges shaped by global food and tobacco industry practices affecting childhood obesity and child well-being. Our study supports the role of regional institutions in providing technical expertise, facilitating peer learning, and setting collective targets, possibly through a “GCC NCD target” that complements national targets.

### 3.7 Lessons learned and implications for national strategy design

**Figure 2:**
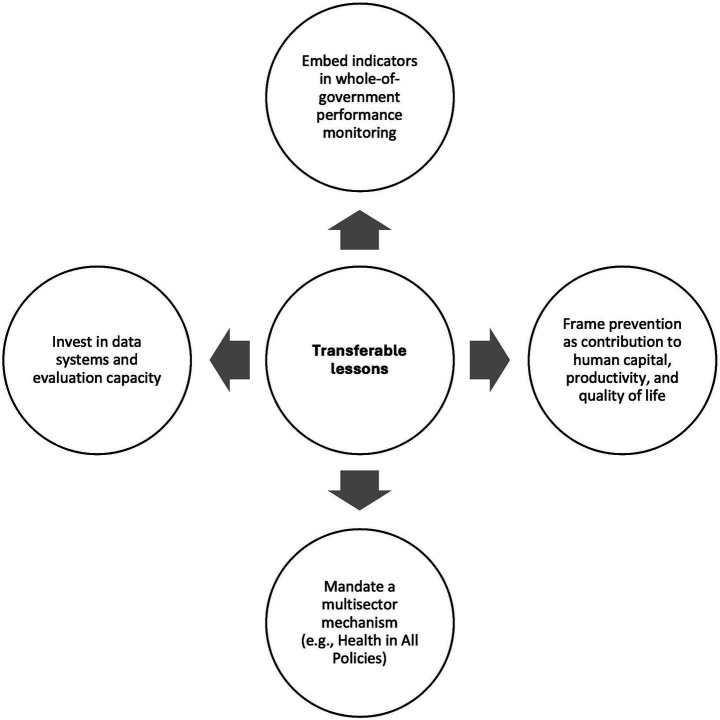
Transferable lessons and design considerations for translating national development visions into NCD prevention delivery.

GCC countries have distinctive governance and planning systems. Our synthesis suggests lessons for countries seeking to use national development strategies, or equivalent whole-of-government plans, to advance NCD prevention (Figure 2). These lessons also highlight the value of regional platforms that can support peer learning, harmonization, and shared technical capacity for multisector NCD action in line with WHO recommendations on multisector coordination and prior GCC regional syntheses (15, 16).

First, visions appear most influential when they move beyond broad aspiration and include a small set of measurable health/NCD indicators that are routinely monitored within central government performance systems. This creates a practical accountability model and encourages non-health sectors to engage.

Second, a “translation model” is needed between vision intent and delivery. Stakeholders’ accounts across countries suggest that multisectoral coordination mechanisms are more likely to sustain implementation when mandates are clear, roles are assigned across sectors, and coordination bodies have sufficient authority and resourcing to request action and follow-up.

Third, many high-impact NCD policies depend on tools outside ministries of health (e.g., guidelines, urban planning and municipal action). Setting these measures as contributions to wider national priorities; such as human capital, productivity, quality of life, or public sector performance may help secure cross-sector ownership and reduce implementation gaps.

Fourth, data and evaluation capacity shape whether visions translate into sustained progress. Where routine data systems are limited, tracking of vision-linked health becomes difficult, and implementation may only be short-term initiatives. Investments in monitoring systems therefore can function as enabling infrastructure for translating vision commitments into delivery.

These lessons do not imply that national visions alone cause policy or outcome change; rather, they highlight design features and enabling conditions that can strengthen the pathway from high-level strategy to multisector NCD action.

Although this study focused on the GCC, several findings may be relevant to other regions that use shared strategies to support national NCD action. The key transferable finding is not that national visions directly improve health outcomes, but that high-level political frameworks can support NCD prevention when they are linked to measurable indicators, multisectoral implementation mechanisms, and routine monitoring. This pattern is comparable to the European Union’s Healthier Together NCD initiative, which supports countries in identifying and implementing effective NCD policies across health determinants, cardiovascular diseases, diabetes, chronic respiratory diseases, and mental health, with emphasis on prevention, early detection, and quality of life (20). It is also comparable to ASEAN’s Post-2015 Health Development Agenda, which includes NCD prevention, tobacco and alcohol reduction, healthy diets, whole-of-government and whole-of-society action, measurable targets, monitoring and evaluation, and digital health (21). However, applicability is context-dependent. The GCC experience is therefore most useful as a set of design principles; align national development strategies with NCD indicators, give regional bodies a clear evidence and convening role, use phased implementation for fiscal and regulatory policies, and establish monitoring systems that support cross-country learning.

### 3.8 Limitations

Our analysis relied on qualitative synthesis of interviews and documents. We added selected public health indicators and implementation milestones from the Gulf CDC mapping report and stakeholder interviews to strengthen the epidemiological and policy context of the findings. These indicators are descriptive and should not be interpreted as evidence that national visions caused changes in NCD outcomes. Quantitative evaluation of NCD trends in relation to vision implementation was outside our scope. Therefore, while we describe associations, such as the timing of policies and outcome improvements, we cannot attribute causality to the visions. Our comparison of NCD-related targets across visions is descriptive and relies on official documents and English translations, rather than a full audit of all planning instruments or unpublished government indicators. Stakeholders may have linked achievements to visions because these are government-driven initiatives, which we addressed by citing concrete policy actions. Implementation constraints may reflect country-specific governance contexts, including variation in institutional capacity, legal frameworks, and resourcing. Nonetheless, the principle that high-level political commitment can support NCD action is likely to hold more broadly.

### 3.9 Future research

Evaluation of vision-inspired policies and NCD outcomes is needed. Mixed-methods studies linking mortality, life expectancy, and risk-factor trends to major vision reforms, while accounting for other influences, would help quantify their contribution. Comparative work outside the GCC could clarify whether observed changes are specific to the vision approach or reflect wider regional trends. Future research should also track GCC-level targets, such as physical activity prevalence, to understand drivers of change, identify governance mechanisms linked to progress, and enable cross-country comparison.

## 4 Conclusion

This study shows that aligning political vision with technical action has been associated with reported progress and plausible pathways in NCD prevention. GCC countries demonstrate how embedding health within national development visions can create a politically authorized, whole-of-government platform for NCD prevention. Countries are well-positioned to translate vision commitments into measurable health gains through cross-sector coordination, data infrastructure, and regional harmonization. These findings align with WHO guidance on Health in All Policies and multisector governance for NCD prevention.

## Data Availability

The raw data supporting the conclusions of this article will be made available by the authors, without undue reservation.
