# Supplementary material for: From economic visions to healthier nations: the role of Gulf Cooperation Council countries national development visions in shaping noncommunicable disease policies
Source: Front Public Health. 2026 Jul 29;14:1899157. doi: 10.3389/fpubh.2026.1899157 (PMC13461473; doi:10.3389/fpubh.2026.1899157)
Supplement: Supplementary file 1 [file Image_1.pdf]

Supplementary Material

1 Supplementary Figure

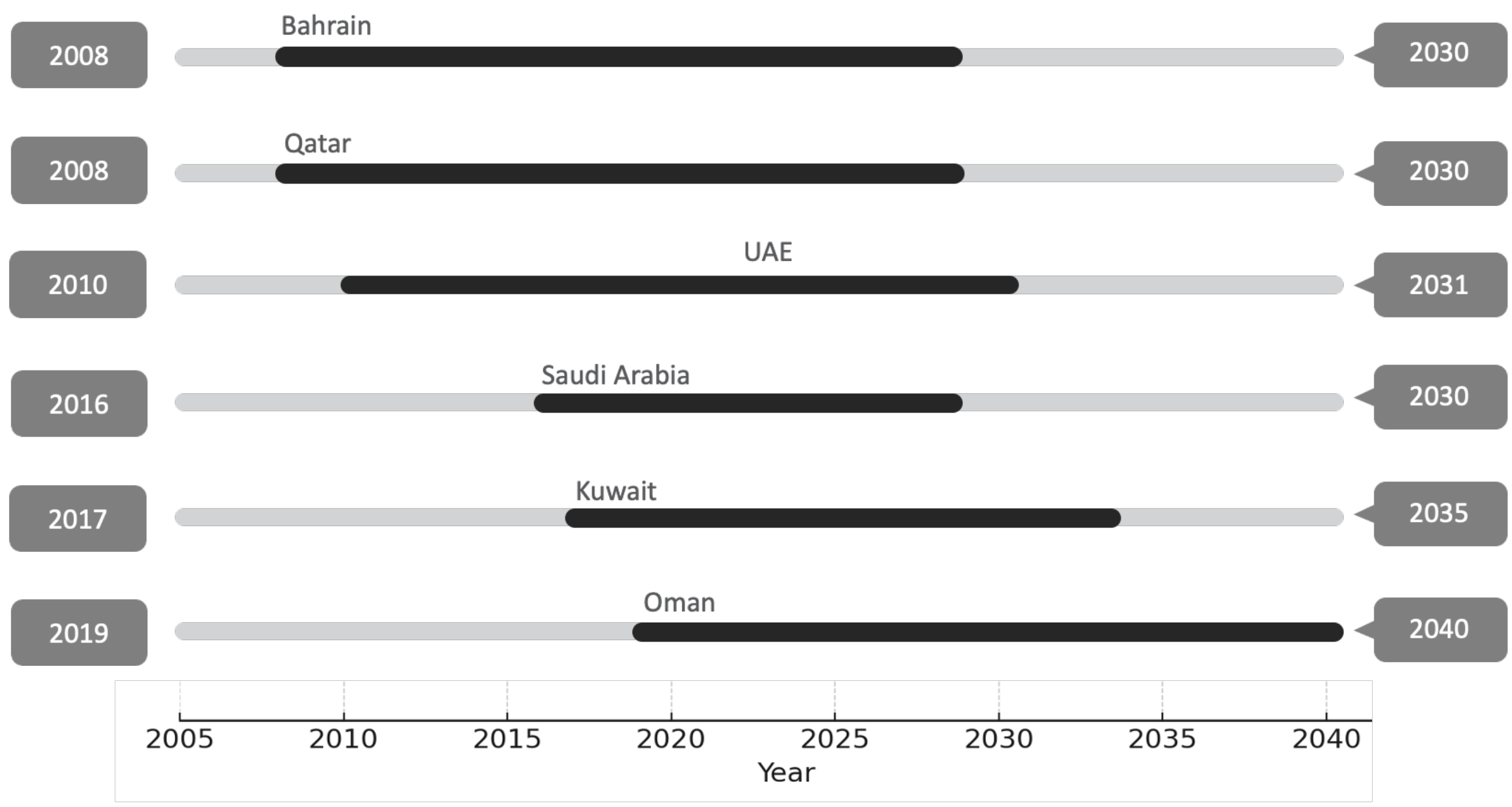

Supplementary Figure 1. Timeline of GCC national development vision frameworks. For each country, the line spans from the vision launch year to its stated horizon (target year).
