# Supplementary material for: From economic visions to healthier nations: the role of Gulf Cooperation Council countries national development visions in shaping noncommunicable disease policies
Source: Front Public Health. 2026 Jul 29;14:1899157. doi: 10.3389/fpubh.2026.1899157 (PMC13461473; doi:10.3389/fpubh.2026.1899157)
Supplement: Supplementary file 2 [file Table_1.docx]

Supplementary Material

# **Supplementary** **Table 1**

**Qualitative Interviews: Interview Guide**

**Project : Policy Mapping on Regional Public Health Policies, Protocols, and Procedures**

| **INTERVIEWEE NAME** |  | | **CONDUCTED BY** | |  | |
| --- | --- | --- | --- | --- | --- | --- |
| **INTERVIEW DATE** |  | **INTERVIEW START TIME** |  | | **INTERVIEW END TIME** |  |
| **POSITION TITLE** |  | | **POSITION DEPARTMENT** |  | | |
| **DESCRIPTION OF POSITION ROLES AND RESPONSIBILITIES** | | | | | | |
|  | | | | | | |

## Introduction

- Welcome the interviewee
  - Thank you for taking the time to speak with us today. The interview is part of a project led by the Gulf Centre for Disease Prevention and Control (GCDC) titled ‘Regional Public Health Policies, Protocols, and Procedures Briefing Report’. The purpose of this interview is to gather qualitative insights into the landscape of Noncommunicable Disease (NCD) prevention and control policies, specifically Cardiovascular Diseases (CVD), Diabetes (DM), and the risk factors for CVD in your country, focusing on institutional roles, government engagement, political commitments, challenges, and the role of regional entities like the GCDC.
- Explain the rationale of the interview
  - Qualitative interviews allow for exploration beyond predefined options, enabling interviewees to elaborate on their perspectives, experiences, and insights. This depth of understanding is crucial for contextualizing data gathered from the objective parts, helping to comprehend not only what policies exist but also why they were developed, how they are implemented, and the challenges they face. Moreover, it can facilitate the identification of success stories, innovative approaches, what is working well, what needs improvement, and the barriers to achieving desired outcomes, which can inform future policy decisions, guide resource allocation, and serve as examples for other regions or countries.
- Explain the interview process
  - Your participation is voluntary, and you can skip a question or stop the interview at any point. All the interviews will be confidential and will not include your name or position title. The information you provide will only be reported in aggregate with other information we collect on CVD, DM, and risk factors for CDV.
  - The information you’ll provide will help us document progress and identify opportunities for improvement. The interview will take no more than **one hour** to complete

| **Do you consent to participate in this interview?** | |
| --- | --- |
| Yes (proceed) | No (thank them and end the interview) |
| ☐ | ☐ |

- Ask for permission to record the interview
  - To ensure we capture all the conversation efficiently, we would like to ask for your permission to record it. Recordings of this conversation will be transcribed into a written document so that we may have an accurate record of the conversation for analysis. The recordings will be accessible to the researchers only and deleted after verification of the transcription.

| **Do you consent to record this interview?** | |
| --- | --- |
| Yes (proceed) | No (do the interview without recording) |
| ☐ | ☒ |

- Thank you very much. We will now begin the interview
  - If you need to think about a question for a little while before answering, please take as much time that you need. Do not feel like you are under any pressure to answer these questions quickly or in a certain way. There is no right or wrong answer.

## **Section 1: Background and NCD prevention and control policy landscape:**

1. Describe your institution's role in the prevention and control of cardiovascular disease (CVD) and diabetes (DM), as well as its risk factors (obesity, physical inactivity, tobacco use, hypertension, high plasma glucose level, dyslipidemia, social determinants of health) or the promotion of health in your country.
2. In your opinion, which ministries/departments/sectors within the government are actively engaged in the prevention and control of CVD, DM, and its risk factors in your country? Describe their roles. For example, the Ministry of Health may be responsible for NCD policy, action plans, guidelines, and healthcare initiatives.
3. Are there other ministries/departments/sectors that are not currently involved but should or could be involved in the prevention and control of CVD, DM, and its risk factors? If yes, how can they contribute?
4. In your opinion, which of the entities provides leadership for the prevention and control of CVD, DM, and its risk factors in your country?

Note**:**

Leadership refers to the provision of overall direction for NCD prevention and control. This includes the responsibility for defining, supervising, monitoring, and managing the national agenda and strategies related to NCD prevention and control. Effective leadership in the NCD involves coordinating efforts across multiple stakeholders, various government ministries, departments, and external organizations to ensure a comprehensive and integrated approach to reducing the burden of NCDs within a country. It also entails making informed policy decisions, allocating resources to address NCD risk factors and conditions.

Coordination means communicating on and aligning actions and developments relating to NCD policies. It could include facilitating regular exchanges between relevant stakeholders, such as a multisectoral committee or working group.

### Political stream:

5. Within the past 5 years (2018-2023), how have political commitments changed in your country regarding policies on the prevention and control of CVD, DM, and its risk factors?
6. Can you provide some information on key past events such as political changes, position statements, or scientific events that have shaped the CVD, DM, and its risk factors action plan in your country?
   a. Specifically, how have healthcare transformations, national visions (such as KSA Vision 2030, Oman Vision 2040, UAE Vision 2021, and Qatar National Vision 2030), and other political changes, position statements, or scientific events contributed to the policy development in your country?

## **Section 2: Current and planned NCD- (CVD, DM, and its risk factors) policies by sector (these should be customized for each sector):**

7. In your opinion, which is the most important CVD, DM, and its risk factor policy in your sector?
   a. During the development of the most important CVD, DM, and risk factors prevention and control policies or action plans, was a consultative process involving relevant stakeholders used?
   b. If yes, please describe the process (which stakeholders were involved)?
   c. Were there any challenges in engaging government ministries or other agencies through such processes?
   d. Was there any private sector involvement in policy development?
   e. If yes, what role did the private sector play in the policy development? (i.e., contribution to policies related to taxation, changing menus in food service establishments, nutritional labeling and reformulation of processed foods, advertising regulations for unhealthy products, smoking cessation programs etc.)
   f. What specific contributions did the private sector make (e.g., data sharing, funding)?
   g. Were there any public consultations or forums held to gather community input?
   h. If yes, how was feedback from the community incorporated while developing the policies?
8. Do you have a current national communication strategy (using mass media) to raise awareness and promote prevention and control of CVD, DM, and its risk factors?

For example:

- Public understanding of how to read calorie information on menus mandated to be added.
- Public awareness of the tax on sweetened beverages, smoking cessation programs, and the harms of tobacco use.
- Knowledge of the public on the importance of regular physical activity.
- Information about hypertension screening and management. (health promotion campaigns, medical camps, etc).
- Public awareness of healthy eating guidelines and reducing salt intake?

9. What are the areas of greatest progress in the prevention and control of CVD, DM, and its risk factors in your sector? Are there specific achievements or improvements that you'd like to highlight? (opportunities)

## **Section 3: Challenges**

These sets of questions are about the challenges and facilitating factors associated with CVD, DM, and its risk factors policies.

10. What have been the most significant challenges encountered by your country in initiating or sustaining national-level approaches to NCD prevention and control in recent years?
11. Based on your experience, please provide up to three recommendations you would offer to another country that is in the process of developing a national policy for NCD (CVD, DM, and its risk factors) prevention and control.
12. What key factors enabled and facilitated the successful implementation of the policies?
    a. What strengths of your policy implementation strategy are noteworthy? (success stories)
    b. Will those strengths serve as models for other regional countries seeking to achieve similar goals?

## **Section 4:  Understanding the role of the GCDC**

13. How can the Gulf Centre for Disease Prevention and Control (G-CDC) support your country and other Gulf countries in the prevention and control of non-communicable diseases (NCDs), (with a focus on strategies and policies)?
14. What are your expectations for the future roles of G-CDC, particularly about policy development, strategy implementation, and overall support for NCD prevention and control?
    a. How do you envision G-CDC contributing to areas such as data collection, technical assistance, monitoring and evaluation, and capacity building to enhance NCD prevention and control strategies?

## **Section 5: Conclusion**

- Summarize the main discussion points.
- Ask the interviewee if they would like to add anything else.
- Thank them for their time.
