# Supplementary material for: From economic visions to healthier nations: the role of Gulf Cooperation Council countries national development visions in shaping noncommunicable disease policies
Source: Front Public Health. 2026 Jul 29;14:1899157. doi: 10.3389/fpubh.2026.1899157 (PMC13461473; doi:10.3389/fpubh.2026.1899157)
Supplement: Supplementary file 3 [file Table_2.docx]

Supplementary Material

Supplementary Table 2. Selected NCD indicators/targets and health governance structures in GCC countries under their national visions

| Country | Selected NCD Targets/Indicators (post-vision) | Health Governance Structures for NCDs |
| --- | --- | --- |
| UAE | - UAE vision 2021 aligned with WHO NCD global targets to reduce premature NCD mortality by 25% by 2025.  It emphasizes the importance of preventive medicine and seeks to reduce cancer CVD mortality by 30% and lifestyle related diseases such as halt rise in diabetes & obesity, 25% reduction in smoking prevalence to ensure a longer, healthier life for citizens. In addition, the Agenda aims to increase the healthcare system's readiness to deal with epidemics and health risks. These was reflected in 2018 by reducing diabetes prevalence from 16% to 11.8% and reducing smoking prevalence to 9%. - The UAE Centennial Plan 2071 is a long‑term national strategy launched in 2017 , Its objective is to make the UAE the best country in the world by 2071, with health, wellbeing, and quality of life as central pillars. - UAE national multisectoral NCD prevention plan and the national policy for promoting healthy lifestyles are aligned with SDGs to reduce NCD premature mortality by 30% by 2030 and promoting healthy lifestyle ( healthy eating, physical activity, and tobacco control), UAE on the track to achieve SDGs 2030. | - **National NCD Committee and Multisector Teams**: Housed under the Ministry of Health and Prevention (MoHAP), a national committee on NCDs was formed following the UAE’s commitment to the WHO Global Action Plan. The interviewee described national multidisciplinary teams for major NCD areas (diabetes, CVD, cancer, etc.) that include *“representatives from all the local health authorities… and from different ministries (Education, Interior, Community Development, Transportation, Municipalities… even Defense medical services)”* (Head of NCD, MoHAP, UAE). These teams jointly develop action plans and interventions, ensuring that each sector contributes (e.g. Interior Ministry on traffic accident prevention, municipalities on healthy food environments). The teams have been active since ~2018 and were recently updated to align with new 2031 goals. - **Health Council**: The UAE has a federal *Emirates Health Council* that coordinates health strategy across the seven emirates (since healthcare is partly devolved). This council, chaired by MoHAP, aligns emirate-level health initiatives with national vision targets, including NCD prevention programs, and shares data and resources nationwide. - **Policy Integration mechanisms**: The UAE uses a *“Health in All Policies”* approach via the Cabinet: for example, a *Quality of Life Council* under the UAE Cabinet includes health and non-health ministers to drive intersectoral initiatives (like active living campaigns, sugar intake reduction) supporting Vision 2031. UAE’s approach tends to integrate NCD objectives into broader agendas such as the National Wellbeing Strategy and National Nutrition Guidelines, with high-level oversight.– - **Public-Private and NGO Engagement**: Recognizing the role of non-government actors, UAE’s national NCD team also involves major private healthcare providers and NGOs. *“Private entities… big hospital chains… are also represented”* in the national partnership for NCDs. Additionally, NGOs (e.g. cancer societies, diabetes association) participate in awareness campaigns and service delivery, under MoHAP’s coordination. This inclusive model leverages all societal players to achieve the vision’s health goals. - **Data and Monitoring**: UAE Vision targets are tracked via an online dashboard of National KPIs. Annual reports on Vision 2021 showed status of each health indicator, fostering accountability. This rigorous monitoring continues for Vision 2031, prompting continuous policy refinement (for instance, when data showed slower progress on obesity, it led to launching a multi-sector National Nutrition Strategy in 2020). |
| Bahrain | - Committed to SDG 3.4: reduce premature NCD mortality 1/3 by 2030. National target to reduce hypertension prevalence; e.g. salt-reduction policy aims for measurable drops in population blood pressure by 2030 (monitoring via national health surveys). - Increase physical activity and reduce obesity (part of National Health Strategy aligned with Vision 2030). | - **Supreme Council of Health (SCH):** oversees national health policy, above the MoH. All major health policies (including NCD prevention regulations) require SCH approval, ensuring alignment with national vision goals. - **Ministry of Health Public Health Directorate:** leads NCD programs (e.g. Nutrition Section for diet-related policy, as interviewee described) and coordinates with other ministries. Under Vision 2030, public health was organizationally separated from treatment services to focus on prevention. - **National NCD Committee or “Health Promotion Council”:** multidisciplinary committee under the National Plan for Control of Chronic Diseases, involving stakeholders from government and NGOs to guide NCD strategy (e.g. diabetes, tobacco). - Multi-sector collaborations (e.g. with education, municipalities) for specific risk-factor initiatives, though these are often project-based. |
| Saudi Arabia | - Vision **2030 health targets:** Increase life expectancy to 80 years; reduce the percentage of adults physically inactive from 86% (2015) to 60% (i.e. 40% exercising weekly); lower smoking prevalence (target: 5% relative reduction/year as per MoH plan); and achieve a 25% reduction in premature NCD mortality by 2025 (aligned with global). - The Health Sector Transformation Program (HSTP) under Vision 2030 includes indicators like reducing obesity and diabetes prevalence (e.g. halt the rise in obesity by 2030), increasing screening rates (e.g. 70% of adults screened for hypertension). Also, specific risk-factor policies: Saudi eliminated industrial trans fats by 2020 and set strict limits on salt in foods.– By 2024, life expectancy had already risen to ~78.8 years, indicating progress toward the 80-year goal, partly attributable to NCD control efforts. | - **Council of Ministers & Vision Realization**: Under Vision 2030, a high-level committee within the Council of Ministers oversees the Health Transformation. The *Ministerial Committee for Health in All Policies* (est. 2018) is chaired by the Health Minister and includes ministers of education, municipal affairs, media, etc., ensuring non-health sectors incorporate health objectives. *“Various ministers whose decisions impact health are on this committee… we discuss all national policies under Health in All Policies”* (Public Health Authority official, KSA). - **Public Health Authority (PHA)**: Established in 2022 (evolving from the Saudi Center for Disease Control), the PHA is an independent entity mandated to lead disease prevention and health promotion policies. According to its charter (Article 4), it develops national public health policies and coordinates multisectoral efforts. The PHA houses scientific committees (for tobacco control, nutrition, etc.) that include multi-agency experts, ensuring evidence-based policy design. - **Saudi Food and Drug Authority (SFDA)**: A key regulatory agency under Vision 2030’s health objectives, SFDA has driven nutritional policy changes (e.g. mandatory calorie labeling, sugar reduction in beverages, front-of-pack labeling). The SFDA works in tandem with other bodies (e.g. with Municipalities for restaurant regulations) and uses platforms like the public consultation portal (Istitlaa) to engage private sector and civil society in policy development. One interviewee from SFDA noted that *“legislation… is issued by SFDA, but we conduct broad consultation… ultimately Vision 2030 is the inspiration guiding us to reduce health risks”*. - **National NCD Program & Committees**: The MoH’s National NCD Program coordinates clinical preventive services (e.g. hypertension and diabetes screening in primary care). Meanwhile, multiple National Committees exist for specific health topics (around 12–16 committees), for example, a National Committee for Tobacco Control, Healthy Diet Committee, etc., each with multi-agency membership. These feed into policy proposals that go through a Preparatory Committee and then the Board of the Public Health Authority or higher authorities for approval. This structured pathway was put in place to expedite policy implementation under Vision 2030’s tight timelines. - **Regional and Local Governance**: At the province level, Health Clusters and local authorities implement NCD initiatives in line with national policy. The Vision 2030 framework encourages local accountability for health outcomes, backed by annual performance agreements. |
| Oman | - NCD policy targets aligned with global goals: 25% reduction in premature NCD mortality by 2025 (per WHO) and SDG 3.4 by 2030, as adopted in the *National Policy for Prevention and Control of NCD 2016–2025*. - Specific national targets in the Multisectoral Action Plan: e.g. reduce physical inactivity by 10%, reduce tobacco use by 5%, halt rise in obesity by 2025 (cited in MoH documents). Vision 2040 reinforces these by calling for *“enhanced quality of life”* and monitoring progress via biennial indicators. - Improve healthy life expectancy (indicator tracked under Vision 2040, though exact target not public; Oman’s healthy life expectancy was ~67 years in 2019). | - **National Multi-Sectoral Committee for NCDs**: established under MoH in early 2010s to develop the NCD policy and action plan. It included representatives from health, sports, education, municipal, commerce and other sectors. This committee successfully formulated a national multisectoral NCD plan and various sub-plans (e.g. a dedicated *National Physical Activity Plan*). *However, the committee’s legal mandate was weak; it operated on voluntary inter-sector cooperation. After COVID-19, the committee and its “teams” became inactive and are now being re-established* (Director of Community Health Initiatives, Oman). - **Vision 2040 Implementation Follow-up Unit**: a government unit that tracks sectoral progress on Vision 2040 goals, including health metrics. Interviewee noted this *“Vision Office”* monitors ministries and *“questions why something hasn’t been done,”* adding positive pressure for implementation (Oman). - **Ministry of Health: Directorate General of Primary Health Care**: within which the Community Health Initiatives Dept coordinates NCD prevention at the primary care and community level. Oman’s MoH also integrates NCD services in primary healthcare (e.g. establishing “healthy lifestyle clinics” and Healthy Cities programs) as part of Vision 2040’s people-centric health approach. - **No separate Supreme Health Council**; health policies are approved through normal ministerial processes, but high-level inter-ministerial engagement is sought when needed (e.g. via Cabinet directives). Oman’s experience highlighted the need for a binding framework (e.g. a law or decree) to ensure sustained multisectoral action on NCDs. |
| Qatar | - Adopted national NCD targets in line with WHO and SDG goals: e.g. 25% reduction in premature NCD mortality by 2025; 30% reduction in tobacco use; 10% reduction in obesity by 2025 (per Qatar Public Health Strategy). - Recent outcomes indicate progress: NCDs cause ~72% of deaths, and Qatar aims to bend this curve through preventive policies (e.g. 30% reduction in salt intake achieved in bread products; sugary drink tax (50%) introduced, tobacco tax 100%). - Increase screening coverage: Qatar has 6 national screening programs (including for diabetes and CVD): target >75% uptake in eligible populations. Also, targets for expanding physical activity and achieving “healthy city” status nationwide (all 8 municipalities achieved WHO Healthy City status by 2022, making Qatar one of few countries with 100% coverage). | - **Ministry of Public Health (MoPH)**: provides strong stewardship, as noted by stakeholders. The MoPH leads policy formulation and has a dedicated Public Health Department for NCDs. It convenes an *“inter-ministerial committee”* on health that includes most ministries, ensuring coordination on policies like road safety (with Ministry of Interior), sugar taxation (with Ministry of Finance), healthy urban planning (with municipalities), etc. This high-level committee has been instrumental in multi-sector laws (e.g. mandating healthy city standards across municipalities). *“We have a committee… including various ministers related to health… [which] assures alignment of health and other sectors”* (Ministry of Public Health official, Qatar). - **Cross-sector policy teams**: For specific initiatives, Qatar forms task forces. E.g., a task force with Ministry of Environment for air pollution control, with Ministry of Education for school health, and collaboration with major semi-government entities (Qatar Energy, Armed Forces) to implement workplace wellness. These arrangements are often under the umbrella of the National Health Strategy implementation committees.– - **Former Supreme Council of Health**: Qatar’s health ministry was previously named the Supreme Council of Health (until 2016), which had broad oversight of health policy. Under QNV2030, that structure evolved into the MoPH but retained high-level leadership engagement. Senior government officials (up to the level of the Prime Minister) have publicly endorsed NCD initiatives, ensuring political buy-in. - **Monitoring & innovation**: Qatar uses advanced surveillance (e.g. national NCD registry, periodic STEPwise surveys) to track progress, and has invested in e-health and digital tools aligning with Vision 2030’s modernization agenda. The MoPH reports on health KPIs to the central planning authorities to demonstrate alignment with the vision. |
| Kuwait | - Aim to improve life expectancy (current according to World Bank 2023 data: 83.19 years) by tackling the four major NCDs which account for ~65% of deaths, while total NCD mortality is ~79% (World Bank 2019). - National development plan includes KPIs like reducing smoking prevalence and improving diet (no public numeric targets in Vision, but health ministry set goals aligned to WHO targets: e.g. halt rise in obesity/diabetes by 2025). - Achieve ≥80% cities with “Healthy City” status (target underway: progressing toward achieving WHO-certified healthy cities in all governorates of Kuwait, with several towns already engaged in the certification process). | - **Ministry of Health (MoH): NCD Prevention and Control Directorate:** oversees NCD strategy and integrates it into the national development plan. MoH leads specific programs (e.g. National Anti-Smoking Program, Healthy Cities Office) that span sectors. - **Supreme Council for Planning and Development (SCPD)**: high-level body that monitors the implementation of *New Kuwait 2035* across all pillars, including health. Health projects (e.g. NCD prevention initiatives) in the 5-year development plans are tracked by SCPD, which facilitates coordination among ministries. This ensures accountability of non-health ministries for health-related objectives in the Vision. - **Intersectoral Partnerships**: e.g. Healthy Cities Committee involves 21 government sectors beyond MoH (municipalities, environment, education, etc.) and 19 within MoH, serving as a *“hub for intersectoral collaboration”* on the social determinants of health. These partnerships are often formalized via memoranda of understanding (e.g. MoH with Environmental Authority for anti-smoking law enforcement).– No separate “Supreme Health Council” in Kuwait; instead, Cabinet and SCPD provide oversight. The Cabinet monitors key health outcomes as part of the government work program, reflecting Vision 2035’s whole-of-government commitment. |

Source: Gulf Center for Disease Prevention and Control. Mapping of NCD Policies in the GCC Region. Gulf CDC policy mapping report, 2025; supplemented by key-informant interviews conducted as part of the Gulf CDC NCD policy mapping project. Indicators are descriptive and do not establish causal effects of national development visions on NCD outcomes.
