# Supplementary material for: From economic visions to healthier nations: the role of Gulf Cooperation Council countries national development visions in shaping noncommunicable disease policies
Source: Front Public Health. 2026 Jul 29;14:1899157. doi: 10.3389/fpubh.2026.1899157 (PMC13461473; doi:10.3389/fpubh.2026.1899157)
Supplement: Supplementary file 4 [file Table_3.docx]

Supplementary Material

Supplementary Table 3. Early outcomes and NCD “evidence of progress” linked to national Visions in GCC countries

| Country | Illustrative progress signals reported in the mapping report and interviews | Reported continuity and monitoring |
| --- | --- | --- |
| United Arab Emirates | Reported reductions in adult smoking and stabilization of diabetes prevalence by around 2020, alongside a small rise in healthy life expectancy, described as consistent with national performance priorities. | Stakeholders emphasized routine performance tracking and continued resourcing for prevention, surveillance, and early detection as part of national agenda delivery. |
| Bahrain | Salt reduction in bread reported as achieving a sizeable reduction in salt content, with high compliance among bakeries, and described as a cost-effective prevention approach. | Phased implementation with monitoring through regulatory and inspection functions was described as supporting durability, alongside leadership and budgeting support. |
| Saudi Arabia | Life expectancy reported as increasing during the Vision 2030 period, moving toward the stated 2030 target. | Health sector transformation financing mechanisms were described as opening new funding streams for prevention and system strengthening priorities. |
| Oman | Prevention oriented implementation milestones reported included physical activity promotion activities plus integration of physical activity assessment into routine primary care electronic records. | Stakeholders described reliance on existing resources and systems to sustain activities, with expectations that future WHO STEPwise approach to NCD risk factor surveillance will reflect changes. |
| Qatar | Premature cardiovascular mortality reported as declining over the last decade, described by stakeholders as occurring alongside intensified prevention aligned to Vision framing. | Stakeholders described Vision 2030 as a framework used to sustain cross sector attention to prevention, including through national reporting by the health sector. |
| Kuwait | Healthy Cities milestones reported included multiple localities registered and several Healthy Cities, plus routine integration of diabetes and hypertension services into primary care. | Embedding prevention-oriented initiatives into national development planning was described as enabling continuity through Cabinet level monitoring and more predictable resourcing. |
| Acronyms: NCDs: noncommunicable diseases; WHO: World Health Organization.  Source: Gulf Center for Disease Prevention and Control. Mapping of NCD Policies in the GCC Region. Gulf CDC policy mapping report, 2025; supplemented by key-informant interviews conducted as part of the Gulf CDC NCD policy mapping project. Indicators are descriptive and do not establish causal effects of national development visions on NCD outcomes. | | |
