# Supplementary material for: From economic visions to healthier nations: the role of Gulf Cooperation Council countries national development visions in shaping noncommunicable disease policies
Source: Front Public Health. 2026 Jul 29;14:1899157. doi: 10.3389/fpubh.2026.1899157 (PMC13461473; doi:10.3389/fpubh.2026.1899157)
Supplement: Supplementary file 5 [file Table_4.docx]

Supplementary Material

Table 4. Public health indicators and implementation examples supporting the vision-linked NCD policy synthesis

| Country | Public health or implementation indicator | Link to vision |
| --- | --- | --- |
| United Arab Emirates | 33% target reduction in premature NCD mortality by 2030; annual monitoring indicators reported to the Prime Minister’s Office; prediabetes campaign reaching more than 13,000 participants | Vision 2031 and Centennial 2071 support prevention, quality of life, digital health, and monitoring |
| Bahrain | Salt-reduction indicators for bakeries; 50% of 670 bakeries in green or orange status; breastfeeding increased to 26%; 85% government canteen compliance | Bahrain Vision 2030 and national NCD action plan support prevention, nutrition regulation, school food standards, and early-life prevention |
| Saudi Arabia | Healthy Food Strategy since 2018; around 25 nutrition-related regulations; PHO ban; WHO certification as free of industrially produced trans fatty acids | Vision 2030 and Health Sector Transformation Program support healthy lifestyle and risk reduction |
| Oman | Physical inactivity 38.6% in 2017 NCD survey; 2025 survey planned; Healthy Cities and Villages; household baseline surveys in villages; physical activity assessment in primary care | Oman Vision 2040 adds monitoring and accountability to existing NCD policy structures |
| Qatar | Eight cities and two educational cities approved as Healthy Cities; 30% salt reduction in bread; stakeholder-reported diabetes decline from 16.5% to 15.3%; e-cigarette sales restricted | Qatar National Vision 2030 and National Health Strategy support prevention, screening, healthy cities, and digital health |
| Kuwait | Nineteen registered Healthy Cities; seven WHO-certified Healthy Cities; one healthy university city; four certified health-promoting malls; development-plan budget and Cabinet monitoring | New Kuwait 2035 and the development plan support Healthy Cities as a cross-sector platform |

Source: Gulf Center for Disease Prevention and Control. Mapping of NCD Policies in the GCC Region. Gulf CDC policy mapping report, 2025; supplemented by key-informant interviews conducted as part of the Gulf CDC NCD policy mapping project. Indicators are descriptive and do not establish causal effects of national development visions on NCD outcomes.
